# Counter-narratives against hardships among Syrian refugee youth and parents

**DOI:** 10.1177/13634615231191993

**Published:** 2023-11-07

**Authors:** Els Rommes, Nisrine Chaer

**Affiliations:** 1 Gender & Diversity Studies, Radboud University; 2 Gender Studies, Utrecht University

**Keywords:** refugees, victim, narratives, memories, identity

## Abstract

The conventional literature and popular media describe the challenges of (Syrian) refugees in terms of their being victims who need to deal with the traumatic events they experienced before and during their flight. Their lack of seeking professional psychosocial help to improve their mental wellbeing is often explained by migrants’ supposed fear of stigmatization. Using in-depth interviews with 10 Syrian refugees in the Netherlands, we show that their main struggle concerns their identity fragmentation as a result of both their displacement and the stereotypical discourses of Muslim/Syrian people as victims or terrorists. In this article, we explore how Syrian refugee youths use strategic forgetting and remembering of both positive and negative memories to reconstruct their (collective) identity. Our finding that Syrian refugee youths use counter-narratives of being strong and competent to deal with their experience of identity fragmentation offers an alternative explanation for refugees not seeking professional help in dealing with their hardships.

## Introduction

Since 2011, conflict in Syria has forced more than 5.6 million people to flee their homeland and seek refuge in other countries (UNHCR, 2019). Syrian children comprise more than half of these refugees (UNHCR, 2019). An uprising against the repressive Assad regime began in 2011, but in the following years much of Syria became a warzone due to the militarization of the uprising, the rise of Islamism, and the regime's exacerbation of the conflict (Yassin-Kassab & Al-Shami, 2018). Many Syrian refugees fled to neighboring countries, although approximately 1 million applied for asylum in Europe (UNHCR, 2019).

Over the past few decades, researchers have explored hardships among refugee youth, with a focus on examining post-traumatic stress disorder (PTSD) and other mental health problems caused by exposure to war and violence in their countries of origin and during their flight (Atallah, 2017; Breslau, 2004; Kim, 2016). Conventional literature and popular media describe the challenges of (Syrian) refugees in terms of their being victims who need to deal with the traumatic events they have experienced before and during their flight. Such mainstream approaches to refugee mental health have also promoted debriefing and exposure therapies as suitable treatments for PTSD (Nicholl & Thompson, 2004); however, many refugees do not seek this kind of professional help to deal with their hardships. Some of the main reasons that refugees do not seek help for mental health problems, as noted in the literature, are that they have a fear of being stigmatized, do not know how to access care professionals, or that the mental healthcare system is too Eurocentric (De Anstiss et al., 2009; Heptinstall et al., 2004; Kiselev et al., 2020; Shannon et al., 2015).

Is this assumption that refugees do not access professional help to mitigate their trauma because of a presumed “ignorance” justified by the experiences of refugees themselves? Scholars claim that refugees tend to more commonly associate their mental health problems with the post-migration period rather than the pre-migration phase (Atallah, 2017; Ryan et al., 2008; Stæhr Mia et al., 2007). Indeed, for many refugees, their symptoms might result from their difficult life circumstances, the role of societal expectations, and their loss of autonomy and sense of control over their lives, rather than experiencing PTSD from their flight. Moreover, it is important not to “over-diagnose” Syrian refugees with clinical disorders (Yalim & Kim, 2018). There is an inherent problem in addressing refugees as being in need of help. Gemignani (2011) claims that constructing a refugee's identity around the discourse of trauma and basing psychological healing on talking about and facing the traumas of the past can be problematic. Trauma becomes an objective and concrete reality that is separate from the refugee's interpretations and the sociopolitical context, and a fixed relationship between the refugee's mental health and their traumatic past is established (Gemignani, 2011). The category of refugee becomes founded on social and power-based discourses that focus on the effects of past sufferings (Breslau, 2000; Kirmayer et al., 2003). Carrying a fixed identity for both the individual receiving the label of “refugee” and for those who perpetuate it, this definition of refugee replicates the stereotype of refugees as victims (Gemignani, 2011) while “aggrandizing the Western expert who defines the problem” (Bracken et al., 1997; Ghorashi, 2005; Harrell-Bond, 2002). Similarly, in their study on Syrian refugees’ mental health, Hassan et al. (2015) argued for the importance of shifting researchers’ emphasis from vulnerability-based frameworks toward resilience- and recovery-based approaches, thereby recognizing refugees as active agents in their lives in the face of hardships.

Building on refugee studies investigating the construction of identities and narration of memories (Brough et al., 2013; Gemignani, 2011; Puvimanasinghe et al., 2015; Ramsden & Ridge, 2013), this article investigates those narrative processes among Syrian refugee youths in the Netherlands. We explore how Syrian refugee youths use narratives to recreate the past and to construct their identity in the present against narratives of exclusion from their home- and host land, and their experience of hardships. We examine the specific role of narrative and memory in the continuous identity constructions among a refugee population that has experienced large-scale displacement and stereotypical media portrayals of the Muslim/Arab/Syrian subject in Europe. This anthropological research asks the following question: How do Syrian refugee youths and parents in the Netherlands use counter-narratives to deal with their hardships?

This article seeks to understand the hardships Syrian refugee youths’ experience, arguing that these are mostly related to problems with identity fragmentation. We also identify the memory-related processes through which refugees create counter-narratives to rebuild their identities by analyzing semi-structured and open-ended interviews with 10 Syrian refugee youths and parents, based on a phenomenological approach (Giorgi, 2009) and a narrative methodology (Riessman, 1993). Building on studies on the construction of identity, meaning-making and the narration of memories (Brough et al., 2013; Gemignani, 2011; Puvimanasinghe et al., 2015; Ramsden & Ridge, 2013), this article is a study of the selective remembering of certain events and the forgetting of others to create counter-narratives. Our finding is that Syrian refugee youths use the counter-narratives of being strong and competent to deal with their experience of identity fragmentation in a context of displacement and the dominant discourse of refugees as victims in need of help, offering an alternative explanation for why refugees do not seek professional help in dealing with their hardships.

## Hardships of refugees

Syrian refugee youths experience sources of distress in three phases: the pre-migration period in Syria, the period of flight, and the post-migration period of resettlement in the new host country. In the pre-migration period in Syria and during the flight, many refugee youth will have witnessed the destruction of their homes, experienced a lack of access to basic services, and endured rights violations or violence, putting them at risk of grave psychological distress (UNHCR, 2013). In the post-migration phase, various sources of distress for Syrian refugees are identified. The first is the loss of and grief for missing or deceased family members and/or material losses, as well as feeling concerned about the safety of family members (Hassan et al., 2015), which lead Syrians to experience feelings of estrangement and yearning for the lost homeland. This also implies a loss of everyday implicit sources of identification, such as having bonds and a position in social networks, shared world views and systems of belief (Silove, 2013), and a shared sense of national pride.

The second source of distress is socioeconomic and legal problems and insecurities. Refugees struggle to adapt to a new society and new social fabric in which they are isolated from larger support structures (Thorleifsson, 2014). In this process, they generally experience social isolation, low levels of social support, language barriers, and school delays (Hassan et al., 2015; UNHCR, 2013; Yaylaci, 2018). Interrelated with these social problems are economic problems: more than three-quarters of Syrian refugee households in the Netherlands were living on a low income in 2016 (CBS, 2018). Added to this are legal difficulties and uncertainties related to asylum-seeking procedures. Scholars such as Laban et al. (2004) and Ghorashi et al. (2018) have indicated that the prolonged duration of waiting and insecurity during the asylum process in the Netherlands is a major cause of distress for asylum seekers. According to (Silove, 2013, p. 243), “unstable conditions (…) interfere with the person's capacity to reestablish a coherent and durable sense of identity, and/or to find consistently meaningful roles.” Moreover, refugees experience a change in the meaning or existence of an educational and professional (future) position, which also impacts their identity and self-esteem.

The final source of post-migration distress is discrimination and attitudes toward refugees in the host country, which impact their social identity. Collective or social identity results from belonging to, identifying with, or being identified with a group, such as refugees, or an ethnic minority group, such as Arabs (or people from the Middle East and North Africa), Muslims, and Syrians. This membership may “provide individuals with meaning, support, and agency (i.e., a positive sense of social identity)” and is positively correlated with health and wellbeing. However, “when (…) group membership is devalued or stigmatized, [it can] become a curse, threatening and potentially harming health and wellbeing” (Jetten et al., 2017, p. 789). Indeed, experiences of discrimination are associated with mental health problems among refugee youth (Beiser et al., 2015; Hassan et al., 2015).

The choice to belong to a group and have a specific (social) identity is, however, limited. Bobowik et al. (2017) and Verkuyten and DeWolf (2002) found that the choice to identify as an immigrant or as a Dutch person was particularly limited for those who were more recognizably part of an ethnic minority group in appearance and language, such as Syrians. People who move country are faced with having previously irrelevant characteristics, such as their culture, nationality, religion, or ethnicity, made relevant for them; for example, a Black person may only be noted as being “Black” once they move to a predominantly White country. Migration is an activity that happens in “discursive fields that push migrants to develop specific views of themselves” (Silvey, 2004). Similarly, Kadianaki (2010, p. 438) describes the “immigrant identity” as “negotiated and constructed in particular social contexts, forged through social representations, dominant social discourses, and specific social structures.” According to Yuval-Davis (2010), such discourses or narratives do not necessarily have to be directly based on what a group is like; rather, stories that are not directly about belonging to a particular group “often relate, directly or indirectly, to the perceptions of self and/or Others of what being a member of such a grouping or collectivity (ethnic, racial, national, cultural, religious) might mean” (see also Silove, 2013).

Which stories or discourses about the group “refugees” dominate in the Netherlands and Europe? Although the emphasis in Dutch migration policies in the past (1970s) stressed the rights and diversities of migrants, in the 1990s, a victimhood discourse took over (Ghorashi, 2005). Migrant women in particular came to be seen as vulnerable and in need of protection (Roggeband & Verloo, 2007). Similarly, in Europe more generally, refugees are often seen as helpless victims of oppression, a discourse that can be harmful for refugees who need to be considered as competent to build a new life (Ghorashi, 2005; Harrell-Bond, 2002). These kinds of stigmatization and (de)valuation in discourses about refugees in society, policies, and in the media mutually reinforce each other. Indeed, media portrayals of refugees are often also negative (Esses et al., 2013).

Added to the vulnerability and victimhood discourse, refugees, and particularly Muslim refugees or refugees from the Middle East and North Africa, are not just seen as people in danger but as dangerous people. “Refugees signal the loss of nation—they have lost theirs and here they come to threaten ours! Compassion and hatred intimately intertwine (….) they embody the violence that created them” (Kumsa, 2006, p. 240). In the aftermath of the terrorist attacks by extremist Muslims in the USA and various European countries, and with the rise of the terrorist group Daesh (also known as Islamic State), the refugee became “re-figured as the potential ‘terrorist’ who surreptitiously infiltrates the space of Europe” (De Genova et al., 2016, p. 27). This image of the Muslim “Other” terrorizing the civilized world became conflated with refugees, particularly with those seen as Muslim (Abbas, 2019, p. 2458). As Abbas wrote, this imposed an even stronger pressure on refugees to “perform a non-threatening ‘refugeeness’ that corresponds to received understandings of the refugee as victim requiring protection.” All in all, two discourses on Syrian refugees dominate the popular imagination in Europe and in the Netherlands. The first is the discourse of terrorism and Islamophobia, in which Syrians are considered to be Muslim terrorists and symbols of threat, while the second is the discourse of victimhood, in which refugees are perceived as subjects in need of saving. Both discourses increase the power differences between refugees and the host communities because they impose on Syrian refugees the image of being in need of “helping.” Regarding a group in society as less and in need of help, either because they are seen as Muslims terrorists or as helpless victims, additionally increases the risk of discrimination.

From this summary, it is clear that although potentially traumatic events are a source of hardship, the circumstances in which refugees generally live in their host country should not be underestimated as sources of distress. We showed how this impacts refugees’ identity, or sense of self. The loss of familiar sources of identification, such as social networks and familiar (cultural and physical) contexts; insecurity about their legal position; changes to one's educational/professional future; and the new way society regards you as a person belonging to a specific, stigmatized social group are all elements of the specific context in which refugees now live, influencing how refugees perceive themselves. In this article, we focus in particular on how refugees deal with the fragmentation of their sense of self, their identity, and their feeling of self-worth. Most of the challenges discussed highlight the need of refugees to reconstruct their identities to enclose their losses, accept their new environment, and find a position for themselves between their old home and their new host culture (Gemignani, 2011; Puvimanasinghe et al., 2015; Ramsden & Ridge, 2013). This is all the more complicated in a context dominated by conflicting and negative discourses on refugees, as well as long periods of insecurity during the in-between position of asylum seekers or through the socioeconomic hardships described above. The particular hardships experienced by refugees therefore push them into positions of weakness and fragment their identity in their host society (Munt, 2012).

## Counter-narratives

When people tell stories, they also live the stories they tell. Through narratives or by leaving out parts of their stories (Bloch, 2018), people exchange memories of events, construct specific memories for groups, and shape their identities (Ramsden & Ridge, 2013). Identity construction entails individual and collective narratives of the self and the other, which may be contextual, situational, temporal, or fractured (Anthias, 2008). It occurs in the direct interaction with the story's recipients and with a wider audience (Puvimanasinghe et al., 2015); hence, identities are constructed through (memory-based) stories of the self, by the group(s) one identifies with, and through the stories of others.

Narrating stories can restore the fragmentations in refugees’ identities, thereby helping them to recreate their identities, find meaning, and regain control over their present lives (Frank, 2013) or, as Herman (1992/1997) puts it, creating and revisiting stories allows people to integrate their fragmented memories of displacement. Refugees use memories to deal with experiences of hardship, loss, and resettlement in the current context, and to restore their sense of self-worth (Jedlowski, 2001; Malkki, 1992; Pittaway et al., 2009). At the same time, because traumatic memories and experiences of suffering can be troublesome, individuals may suppress thoughts related to those events (Herman, 1992/1997). Creating narratives is also a strategy of leaving out some parts of the stories in order to move on, live in the present, and focus on the future instead of the past (Bloch, 2018). Silence has been proven to be a tool used to protect the self from the impact of trauma and as a device for newly resettled refugees to reconstruct one's identity (Ghorashi, 2008; Puvimanasinghe et al., 2015; Tankink & Richters, 2007). In their study on refugee women and mental healthcare in the Netherlands, Tankink and Richters (2007) showed how silence can be considered as a coping strategy for dealing with traumatic memories. They explain that memories are subjective processes that are deployed for healing, blaming, or legitimating. In this case, silence can be used as a way to not remember, or as a way to actively forget. In this sense, memories are a tool for creating new meanings of both the past and the present. As they remember, forget, construct, and reconstruct past experiences, refugees engage in a process in which the past is constantly selected, filtered, and reshaped depending on the needs of the present, both individually and collectively (Jedlowski, 2001; Malkki, 1992).

Tankink and Richters (2007) argued that the recollection of memories is not only an individual and internal psychological activity, but simultaneously a collective one. Similarly, in his study on resilience and trauma among Palestinian refugees, Atallah (2017) underlined the importance of conceptualizing resilience as weaving traumatic experiences into larger stories of strength within collective legacies and non-individual forms of agency. Whereas Eurocentric cultural conceptualizations of resilience often focus on individual resilience or the ability to “bounce back” after traumatic events, in many war-torn societies, including the Syrian, there is more emphasis on collective resilience, where the family or community is the main source of strength. Moreover, Syrian refugees share being faced with “tension between their own self-image and the image of them produced by the dominant discourse” (Ghorashi, 2005, p. 185), and so we also focus on the collective resilience as it is shown in the alternative narratives Syrian refugees produce against the dominant discourses.

To underline that the narratives produced by refugees are an empowering alternative against dominant discourses, we use the word “counter-narrative” rather than “narrative.” We follow Puvimanasinghe et al. (2015) in their definition of counter-narratives as narratives that help refugees to regain control over their lives and reconstruct their identities both on the individual and the collective level. Counter-narratives work in two ways. The first is that they serve as a strategy for a refugee to create self-empowering narratives of their life journey, as we described above. The second is their use as a narrative that challenges dominant narratives in society; for example, the view that refugee youths are traumatized, psychologically impaired, or a burden on the host society; or the view of Syrian/Arab/Muslims as violent terrorists. Counter-narratives can collectively contribute to challenging the dominant narratives in mainstream society regarding refugees’ experiences (such as refugees being weak, incompetent or violent, and in need of help) and to constructing alternative narratives of empowerment and resilience (Gemignani, 2011; Ghorashi, 2008; Kadianaki, 2010; Puvimanasinghe et al., 2015). We consider counter-narratives to be specific forms of identity construction that deal with identity fragmentation for individuals and that shape alternative group identities at a collective level.

## Methodology

### Participants

During the period ranging from February to April 2018, we conducted individual qualitative semi-structured interviews with four Syrian refugee youths (aged 18–21) and six parents of children under 18 in the Netherlands (Tables 1 and 2). We chose these age groups because children and young adults are at a phase in their lives during which identity construction is crucial (Erikson, 1968). As a result, it is easier to observe the process of identity construction in these age groups, and the way they deal with hardships can also be expected to have important consequences for their social development. For ethical reasons, we did not want to interview children younger than 18 years of age, so we interviewed their parents. These interviews were somewhat harder to analyze, as they focused on the parents’ perceptions of their children’s struggles, and how they tried to help their children to deal with their hardships, rather than the experiences of the children themselves.

**Table 1.** Overview of the characteristics of parent participants.

Parent (pseudonym)	Refugee status and children	Age	Sex	Education
Ramy	Arrived in 2015, refugee status since 2016. Parent of two children (both 1–6 years)	26–35	Man	Middle-school
Amal	Arrived in 2014, family reunification. Parent of one child (1–6 years)	26–35	Woman	University
Dina	Arrived in 2015, refugee status since 2016. Parent of two children (one below 1 year, one 11–16 years)	36–45	Woman	University
Maria	Arrived in 2016, family reunification. Parent of two children (both 1–6 years)	26–35	Woman	Middle-school
Mohamed	Arrived in 2014, refugee status since 2014. Parent of five children (one 16–21 years, two 11–16 years, two 6–11 years), all born in Syria	36–45	Man	Middle-school
Kinda	Arrived in 2015, refugee status since 2016. Parent of three children (one 1–6 years, one 6–11 years, one 11–16 years), all born in Syria	36–45	Woman	Middle-school

**Table 2.** Overview of the characteristics of the youth participants.

Youth (pseudonym)	Refugee status	Age	Sex	Education
Safir	Asylum seeker since 2015, received negative decision, waiting for appeal decision	18–21	Man	Middle-school
Nizar	Arrived in 2016, refugee status since 2017	18–21	Man	High school
Abou Skandar	Arrived in 2014, refugee status since 2015	18–21	Man	Middle-school
Adam	Arrived in 2015, refugee status since 2015	18–21	Man	High school

The participants were born and used to live in big cities in Syria, such as Souweida, Damascus, Aleppo and Homs, although one participant was born and lived in Kuwait. Most participants currently live in major cities and towns in the Netherlands, although one participant lives in an asylum-seekers’ center. We recruited participants through the personal contacts of the second author, who followed a Dutch integration course in which many Syrian refugees were enrolled; through the list of attendees to a Facebook event that commemorates the victims of a massacre in Syria; and through a Facebook group concerned with the Dutch civil integration exams.

Our method of finding participants for our study resulted in our finding refugees with relatively high levels of education, who may think more positively about professional healthcare. In addition, our recruitment method left out refugees with problems that are so serious that they do not follow Facebook groups, attend integration classes, or respond to an invitation to be interviewed. Nevertheless, we do feel the families and youth we reached provide a good overview of the types of problems encountered by young Syrian refugees and their ways of dealing with them. All of the participants were struggling with their wellbeing, and several experienced more serious mental health challenges with which professional mental healthcare could support them, including the use of alcohol and drugs, experiencing very strong emotions, and having concentration problems. Nizar, for instance, described how, after arriving in the Netherlands, he had serious sleeping problems, “no appetite,” and experienced anxiety. Mohamed described how one of his children had stopped growing, another “pees her pants regularly,” and one of his sons sees a counselor because of aggression problems.

Two of the children of the participating parents had received professional help, which their school had encouraged them to take, and one of the young adults had spoken with a (Dutch) therapist. Several parents and young refugees personally knew friends/family who had recruited professional help, and all said they would “not hesitate for a second” to seek professional help for their mental health problems if they felt they would benefit from it. Several recognized that there could be a taboo on getting mental healthcare, but had not experienced that taboo themselves: “you know, most people from the Middle East have these ideas. They think that mental health patients are crazy or something, or have a really big problem. They don’t understand that mental health problems are just like physical health problems: you need help for it,” Amal explained. Although the interviewed refugees recognized that going to a therapist might carry a stigma for some people, they did not seem to experience such a stigma themselves. Similarly, Hassan et al. (2015, p. 28) wrote that the Syrian refugees they studied did not seem to experience a stigma: “numerous health practitioners working with refugees from Syria (…) have noted that (…) attitudes to MHPSS [Mental Health and Psycho Social Support] services are rapidly changing as a result of the shared experiences of violence, loss, and displacement, which tends to lessen the stigma surrounding mental health problems”. To summarize, the (parents of the) youth we studied did experience problems with their mental wellbeing, knew professional help exists, and did not experience a stigma in obtaining help. Despite this, they did not recruit help themselves.

### Approach

In this study, we combine a phenomenological approach (Giorgi, 2009) with a narrative methodology (Riessman, 1993). A phenomenological approach investigates the meanings that individuals attribute to their lived experience in a context-specific and non-universal epistemology (Giorgi, 2009). The phenomenological approach, which focuses on the meanings of an experience, the psychological state of the participants and the content of their conscious expressions (Bartholomew et al., 2015; Giorgi, 2009), is used in this study to understand narratives and the meanings that participants attribute to stressors and to processes of dealing with stressors. Narrative methodology (Riessman, 1993) is useful for analyzing the narratives of the past and the present and understanding how identities are constructed through narratives and counter-narratives.

### Data collection

The interviews were based on semi-structured and open-ended questions and lasted between one and four hours. Participants were asked to describe their experiences in three different phases: (a) the pre-flight living or parenting in Syria before, during, and after the Syrian uprising in 2011; (b) the flight and forced migration; and (c) after resettlement in the Netherlands. The interview questions focused on psychological challenges and (how they dealt with) their thoughts and feelings about these challenges. For example, we asked participants to narrate their concrete experiences of the stressors and challenges, and to focus on what they felt and thought and on the meanings they attached to practices they use to overcome the stressors and to deal with memories (e.g., “what are the difficulties that you or your child experience in the present/resettlement phase?”; “how did you feel in the past/period of fleeing?”; “give a concrete example of how you deal with memories or with distress”). Follow-up questions were tailored to each participant (e.g., when a participant answered “I sometimes go for a night walk to distract myself from negative thoughts,” we asked “can you tell me more about what you feel when you go for a walk at night?” or “what does it mean to you?”). The interviews were conducted in Arabic, audio-recorded, transcribed, and translated to English by the second author, who is a native Levantine-Arabic speaker.

This study was submitted to the Ethics Committee Faculty of Social Sciences at Radboud University (ECSW-2018-100) and conformed to the requirements. Because of the potential vulnerability of the target group, various measures were taken to support the participants. All participants were given several days between receiving an information letter about the research and giving consent. The choice of location (e.g., their home, a café) depended on the participants’ preferences. Before the interview, the study was verbally explained to participants and a written informed consent form, with information about the study, the right to withdraw at any time during or after the interview without reason or comment, confidentiality, length of interview, risks, and institutional contact details, was provided and signed. Because of the potentially traumatic or triggering content of the interviews, provision was made to address the distress that may have been caused by the interview. To ensure the privacy of the research participants, personal data were anonymized, and the key and data were encrypted, password-protected, and temporarily stored in the server of Radboud University, accessible only by the two authors. Audio-recordings were deleted directly after being transcribed.

### Data analysis

The transcripts were analyzed using the phenomenological approach (Bartholomew et al., 2015) and the narrative approach (Giorgi, 2009). We therefore reviewed the transcripts to determine the meaningful statements or meaning units, which are “parts” of a participant's description of a phenomenon and are used as coding schemes. The authors examined the narrative descriptions, specifically the meanings and feelings that participants attributed to stressors and to memories, as well as the practices of dealing with stressors and memories. Both authors read the transcripts several times, identified similar meaning units in the formulated narratives, and cross-checked these with each other. Examples of the codes include “distracting oneself,” “not dwelling too much on the past,” “experiencing racism,” “feeling strong,” “identity,” and “memories of the past.” The codes were grouped into two themes: “the challenges and realities of being a Syrian refugee” and “how to deal with stressors,” which falls under “the use of empowering memories” and “the use of silence and not dwelling too much on the past.” Finally, the authors jointly analyzed the meaning units contextually and with attention to the overall experience and responses of the participant to identify the links between the found themes, which provided an understanding of how memories and narratives work for participants’ identity reconstruction.

## Findings

In the first empirical section, we discuss the difficulties and challenges that Syrian refugee youths face, which make them feel weak and which we read as a form of loss or fragmentation of identity in the present. In the subsequent two sections, we explain how they use memories to reconstruct identity and to deal with these hardships, using empowering memories and not dwelling too much on the past for their identity construction.

### Hardships faced by Syrian refugee youths

Our research participants recounted several difficulties they experienced in the present, among which stereotypes, difficulties in studying, and structural problems were the most common. Kinda, a mother of three children, stated:

My third son was eight months old when he arrived in the Netherlands. Because he was making a gun shape as he was playing Lego with a Dutch child, they thought that he was performing terrorist acts and they decided to put him under supervision. But why? Your children also play with guns, and the store is full of war games, right? Whether it is a water fight, or electronic or regular, or on a PlayStation. Why is it the case that if it's a Syrian or Arab child you call him terrorist? (Kinda, mother of three)

Kinda reported that her son has experienced negative stereotypes because he is Syrian; he is perceived as a terrorist. This is an example of how Syrian refugees experience discrimination based on their cultural identity and how their reality of having a child being put under supervision is influenced by images of Syrians being a threat. Mohamed had a similar experience to Kinda. When the school told Mohamed, a Syrian father of five children, that “your son is violent; you need to treat him,” he refused, but not, as he later said, because he had a problem with or experienced a stigma about using mental health counselors. Rather, he refused because he felt that his son was facing injustice and was being treated unequally to his peers because he is Syrian. This stereotype of Syrian children being dangerous operates in parallel with the image of them as victims in need of help. Importantly, both images of Syrian cultural identity encompass a forced offer of help in treating his assumed violent nature.

Nizar, a young Syrian refugee, also described how he had to deal with negative stereotypes, in his case surrounding being a refugee:

When you go to the municipality, or to the organizations that help you to find work, these people have started to have certain stereotypes about Syrians. They tell you that you need to work in KFC and McDonald's, you know? (Nizar, 18–21).

Nizar criticizes Dutch institutions and policies because they consider refugees to be low-skilled laborers. He addresses how refugee policies in Dutch institutions overemphasize the importance of quickly finding a job and of learning the Dutch language. Nizar focuses on the expectations and stereotypes about refugees in the Netherlands as uneducated and incompetent, which are discourses that influence the construction of their identity. He seems to suggest that important elements of refugees’ identity are lost because of the label of “refugee” imposed on them by society and institutions. He continued:

My friend is 30 and he is a painter. He only speaks English here and he doesn’t know Dutch at all, but he must do the integration course certificate in six months. So, will [my friend] go work in KFC or McDonald's? No way! He makes art exhibitions and sells his art. There are many people like that, who cannot study language because they already have a career. (Nizar, 18–21)

In this last quote, Nizar makes it clear that the difficult present of his friend is a result of a major part of his identity, being a successful painter, not being seen or respected because of the generic policies and stereotypes, which treat all refugees as a homogenous category without any regard to their individuality. This articulation of difficulties due to stereotypes (which can also be internalized) can also be discerned in the statement of Adam, a young Syrian refugee:

The feeling of being a refugee is something very unpleasant; now I am a refugee and I feel weak because I am a refugee. I left my country… And (…) I experienced racism (…) When one experiences a racist incident, he feels that the people in this country hate him. (…) It just stays like a burn in the heart. (…) When someone tells me “you are a refugee,” he makes me feel that I am not welcome in this country, and this makes me feel weak. (….) I have to put more effort into proving myself than a Dutch person, in studying, work, not being weak, not becoming depressed, not being vulnerable. (Adam, 18–21)

Adam explains that experiencing racism and the stereotype of being a “vulnerable” and “unwanted” refugee, together with being displaced, makes him feel weak. His collective identity as Syrian is replaced with the collective label of refugee.

Similarly, Dina explained how she empowers her son to deal with racism in Dutch society. She told us how she explains to her son that:

Some people are ignorant; others are not open minded and do not know that we come from ancient civilizations. I don’t want to praise our cultures, but I want to say that we are not a bad people; we are not Islamists. I am talking about myself and my family. (Dina, mother of two)

Like Adam, Dina struggles with the label she feels is being imposed on herself and her son. She brings up a collective identity of coming from “ancient civilizations,” which shows her need to build some self-esteem or pride in her son. She feels she needs to defend her son against being seen as an “Islamist,” which in many western societies, including the Dutch, is associated with aggression and terrorism.

The quotes above show the importance and detrimental effects of displacement and particularly of (internalized) stereotypes and discourses of Syrian refugees as weak and/or violent; however, socioeconomic and legal circumstances can have similar effects on refugee identity. In explaining his feelings of weakness, Safir, a young asylum seeker, focused mainly on these kinds of problems:

The amount of money that we take from [the Central Agency for the Reception of Asylum Seekers] (…) literally doesn’t allow for more than food and drink, one return bus ticket, and tobacco. This reality will slowly but surely make your social life deteriorate. For example, if I want to go out, it's limited. I have to count the exact number of times I can take the bus each week. The [refugee] camp is very far, all camps are located in the middle of nowhere. Do they do this on purpose? I have no idea. (Safir, 18–21)

Safir evokes the structural socioeconomic problems related to being an asylum seeker living in a remote asylum camp, which he considers unjust. His description of the deterioration of his social life, and of living “in the middle of nowhere” evokes his experience of displacement and loss of identity.

In the next section, we examine how Syrian refugees use their memories of competence as counter-narratives against the hardships of the present described above.

### Using counter-narratives

#### The use of empowering memories

Adam, who revealed in the quote above how the label “refugee” makes him feel weak, recounts his memories of studying:

Now that I am here [in the Netherlands] I am constantly failing [at school] because I have been out of school for four years. In Syria, I was smart without needing to study, or I would study for a short time and pass. (Adam, 18–21)

Contrasting the current difficulties in studying, he points out that he used to have good grades without needing to study. His memories of the past remind him of the times he felt intelligent, when the characteristics of his identity that made him feel competent were not lost. Those memories are counter-narratives of competence that, together with pointing to circumstances he has faced (being out of school), seem to work as ways to re-establish his sense of self-worth.

Furthermore, in several interviews, the notion of fear and its expression in the present and the past were a recurrent theme. Dina described how her teenage son Amr deals with challenges:

My son suppresses his feelings a lot. He doesn’t like to show them and he doesn’t like it when you show him feelings in return. That's the result of the war. He says, “you should not be afraid.” He lived in the war and dealt with things by himself; he decided to not show his feelings, not be afraid. [He says], “you should not, you should not, you should not [show fear],” and he does that to survive.” (Dina, mother of two)

Dina describes how her son does not express emotions of fear or vulnerability. Her son, who has been through traumatic events in Syria, uses a coping strategy to avoid emotions. Dina explains that, because he experienced trauma and violence, he developed a strategy to protect himself as a survivor, which is his affirmation of his lack of fear and his desire to solve things by himself. Later in the interview, it becomes clear that his “avoidance of emotions” comes primarily from “his need to protect himself,” at least according to Dina. She continued:

Even here [in the Netherlands] he tells me, “the kids here are soft and not tough. We are the generation of war, we are different, we are men. We are not like [Dutch boys].” So these children lost a big part of their childhood and you can see it in their behavior. The 7-year-old kid behaves like a 20-year-old. (….) They are the generation of war. (…) It's a different culture (….) I am against generalizing, but that's (…) what we teach our children: “don’t cry. Men don’t cry.” We want to give them motivation and empowerment, but in reality you are teaching him to suppress his feelings. (Dina, mother of two)

Here, Dina explains how this tendency to suppress emotions, behave like a 20-year-old, and act like a strong and fearless survivor is something that Amr learned as a child of his culture and as member of the “generation of war,” and he continues to practice this in the Netherlands. Furthermore, Amr's affirmation of the toughness of his war-impacted Syrian identity, juxtaposed against the softness and emasculation of Dutch boys, seems to be a strategy to portray himself and his Syrian peers as strong and fearless. It is plausible that his recourse to masculine ideals of strength in describing himself as part of a group and generation that has survived the war is a counter-narrative to the popular image of Syrian children as helpless victims. In other words, his strategy of self-empowerment operates through the creation of a counter-narrative of collective fearlessness against the present view of Syrian children being fearful victims of the war.

For a considerable number of research participants, this process of creating counter-narratives of strength was evident. Abou Skandar, a young Syrian refugee, described how his flight from Syria and his trip across the sea was dangerous. He said:

I always say yes when there is an opportunity [to experience adventure]. I would always be homeless, sleep on the streets. (…) [The flight from Syria] made me stronger. I learned to be more patient, to adapt. (Abou Skandar, 18–21).

Similar to Amr, Abou Skandar talks about his feelings of strength as a result of (his memories of) hardship. Abou Skandar even goes one step further, saying he desires to relive his past dangerous situations because he perceives his flight from Syria was a phase that provided him with the qualities of strength, patience, and adaptation. Hence, his memories, in particular those of dangerous situations, are sources of identity that allow him to recognize who he was and who he has the capacity to be. They support him in positioning himself as a strong survivor rather than a weak victim of war or as a refugee in need of being saved. Adam described his challenging flight from Syria in similar positive terms:

My life was not really difficult. I had more freedom because I was facing more difficulties, so my heart was tougher. Fear increased later [in the Netherlands] because there was stability. (…) Things did not used to scare me before because I was facing them, and knew how to face them, but now (…) the standard of fear has changed. Before I was ready to face such a [dangerous] situation. (…) For example, crossing the sea was dangerous and we saw a lot of drowned people on the way. We spent three days without food or water. (Adam, 18–21).

He emphasized that he experienced his flight and the hardships he endured as something that made him strong: “my heart was tougher.” Adam brings back the empowering memories of his past when he experienced strength, competence, and fearlessness in the face of dangerous circumstances. He proceeds by saying:

So now when I think of what I did [in the past], I don’t understand how was I able to go through all of this. If I think of it now, I think [the things that happened to me] were scary, but in the moment I was not scared. (Adam, 18–21).

In this quote, he describes that now he is no longer under the imminent threat of death or physical violence, he feels confused and he does not understand why he is more afraid in the present, at a time when he experiences stability, than before when he faced grave hardships. By insisting that he was not scared, he is accessing a pleasant memory of not being a fearful person, even in the face of danger. This is an example of fragmentation of identity; specifically, not being able to merge the old and the new self. His individual and collective memory (“we”) serves as a counter-narrative to being seen as, and seeing himself as, a fearful person today. His experience of the present is thus not only influenced by the pre- and post-migration stressors, but also by his self-perception, which is mediated by recourse to selective memories.

Abou Skandar stated:

As you know, we went through a difficult phase and had to leave. (…) I now have my residential status, my life is here, I am building it here, I started school even though I am delayed. I know that I want to live here, and I have to study and work. (…) The beginning was difficult. Many people are afraid to take a rejection for asylum. Thank God I made it. It decreased the stress and made me think about how to begin my life. I have to begin my life here. (Abou Skandar, 18–21)

Like Abou Skandar, many respondents reported the challenges of the present together with the empowering memories of the past, simultaneously extending their statement of their future wishes to start their new life (or for their children to start it) as soon as possible to compensate for the lost years. This tendency to juxtapose hardships and empowering memories into a willful act of positive change demonstrates that recalling the narratives of the past indirectly serves to reconstruct strength and self-worth in the present. Perceiving the future positively is again a way to show strength and competency.

To conclude this section, it is clear that the evocation of toughness by our participants through their selection of empowering memories, as well as through their enumeration of hardships and their strength in dealing with them in the past, may be a coping strategy to deal with being seen as weak in society. The simultaneous reference to their strength and “competent” identity in the past, and the evocation of confusion over current fear or refusal to express fear is a mechanism of redefining selfhood. Longing for a period in which they had qualifications and an identity they are proud of, such as knowing how to deal with difficult situations or their capacities of adaptation, patience, cleverness, and competence, implies they find it difficult to accept the position of perceived weakness in which they now live. It also highlights how the interconnections through which the individual's experience of distress and loss of identity in the present, and the ways of silencing it, are being constituted by the memory of survival in the past. Instead of victimization, they are creating a dynamic state of being a survivor that attempts to restore their sense of self-worth in their present reality.

#### The use of forgetting and not dwelling too much on the past

In this second sub-theme, our findings indicate that our participants engage in a second process of reshaping the present self and reconstructing self-worth. Seemingly oppositional to the process of longing for the past or bringing back empowering memories discussed above, this second process is characterized by the tendency to avoid memories that negatively impact one's identity and not dwell too much on the past. As with the first sub-theme, exploring this second sub-theme provides a deeper understanding of how Syrian refugee youths engage with memories to counter the experience of identity fragmentation.

Many of our respondents insisted that they perform a strategy to forget painful memories. Kinda explained how this process is important for her son:

I don’t talk to [my son] about [the past] or remind him, because if there is something sleeping inside himself, I don’t want it to get out. He doesn’t need it. (Kinda, mother of three)

Kinda believes that the bad memories are “sleeping,” and that her son must bury them rather than being reminded of them. This suggests that, for Kinda, avoiding the narration of painful past events is important.

Adam talked about a similar strategy to deal with current hardships:

I don’t think about [my problems] too much. I don’t like to put negative energy in my body. (…) If I have a problem with a Dutch person or another Arab or whatever, and I am bothered by it, I don’t think about it a lot. I forget it directly, that's it/ (…) I remove it from my head directly. (Adam, 18–21)

Adam recounts that he actively tries to forget unpleasant incidents. The way he phrases this process shows how he regards himself as being in control of his memories and emotions, as well as being capable of deciding whether he will remember a positive or negative event. Arguably, this feeds into the counter-narrative fostered by many of our respondents: of being strong and capable, and in this case of being active and in control.

Seeing memories as something you can actively control was also clear in the next example. When asked about her views on how Syrian children deal with traumatic memories, Amal, a Syrian mother, explained:

If you come from a bad experience and you also face loneliness, then the bad memories keep on repeating themselves; nothing is really changing to make you forget or to replace these bad memories. But if you treat the child in this new situation, the child can forget and doesn’t stay stuck in the same circle. (…) The root of the problem is the bad memories, and treatment can make him forget and protect him from being lonely. So, we must do activities with him, make him frequently meet people. But the thing is, he can forget fast because children are flexible when they are young. He only needs a suitable environment. (Amal, mother of one)

Amal stated that memories of the Syrian war should be forgotten because the bad memories themselves are a problem. Like Adam, Amal regards the forgetting of bad memories as something that can be done and, in this case, to which the environment can contribute. For her, the process of forgetting can happen when a child is distracted, engages in activities, and meets people.

Safir, a young asylum seeker, recounted the strategy he uses and the difficulties he encounters in applying it:

Sometimes I forget and sometimes I can’t. Of course, because I have a lot of free time I think a lot, like about who I was and who I became. And the more I think like that, the more memories come back. I’m trying to forget, because from what I hear, it is not good to stay with your memories, but there is nothing that is making me forget them; my life is empty now. (…) Overthinking is bad, to want these memories to come back. (Safir, 18–21)

Safir states that he thinks about the good memories of who he was and how full his life was, yet to cope with the sense of powerlessness and emptiness resulting from the length of the asylum procedure (more than three years of waiting) and being stuck in the asylum camp, he tries to avoid dwelling too much on the past. Like Amal, he also believes that having social support would help him forget or be distracted from his memories, which indicates that the process of selectively suppressing memories is influenced by the creation of identity and the building of self-worth. Interestingly, for Safir, the bad memories that could interfere in daily life can be of either “good” or “bad” events. Although good memories are empowering, they can also make him feel weak, indicating that it is not the type of event that determines how the memory is experienced, but rather the hardships of the present. In the case of Safir, the hardships of the present are due to the ongoing structural violence of the asylum procedure, which makes him feel empty and unable to distract himself.

Abou Skandar narrated a comparable experience:

I don’t exhaust my mind. (…) Some memories you should forget and some not. For example, the good memories in Syria with my friends, or the memory of my brother dying… that's difficult … but I say to myself that it's not good to keep on thinking. (…) I should think of the future, and try to forget. If I keep on thinking I will be depressed, so I try to distract myself; for example, I study or watch something, so I don’t remember and become depressed. (…) I try to think of the future and the things I like to do instead of making myself tired. (Abou Skandar, 18–21)

Abou Skandar explains the difference between good memories and bad memories, and he emphasizes that one should not dwell too much on either of them, and should instead forget them by distracting yourself. This strategy helps him to move on and to reconstruct his identity from weak to powerful. What is crucial in this quote is his assertion of the importance of thinking of the future, which indicates that his strategy of forgetting should not only be read as an attempt to erase the past, but also as a way to reshape his present and construct a stronger future.

A similarity in many of the narratives about forgetting is that the refugees stated that they need to be able to be distracted in order to do so. Similarly, Renkens et al. (2022) showed the importance of being able to find distraction as crucial for refugees. As we discussed in the Introduction, and as became clear in the stories of our respondents, many of them feel isolated and lack a socioeconomic context in which their “forgetting” is facilitated. Living in a context without meaningful activities to engage in is tough for anyone, but for refugees whose sense of self(-esteem) is under threat and who no longer want to ruminate on their past, this is even harder. The “forgetting” that our informants discuss does not seem to be an example of a pathological psychological avoidance of memories because many of the avoided memories are not of traumatic events, nor do the memories seem to be intrusive. Furthermore, the refugees do not seem to experience a rebound effect from pushing them away. Instead, the type of forgetting for these refugees seems to be more about “not ruminating.” They try to distract themselves from “focused attention on the symptoms of one's distress, and on its possible causes and consequences, as opposed to its solutions” (Nolen-Hoeksema, 1998). Distracting oneself from ruminating is generally considered an effective way of coping by therapists; however, further exploration of individual cases of distracting oneself and “pushing away” bothersome memories is needed to determine whether it is a (successful) way to avoid ruminating, rather than a counterproductive attempt to deal with (symptoms of) PTSD.

## Discussion

Our findings challenge the notion that refugees suffer from trauma mainly acquired before or during their flight by showing the relevance of post-migration stressors. Although the study and treatment of trauma in refugees using traditional western approaches provides insight into the relevance and treatment of traumatic events and symptoms of PTSD, it is also important to capture elements of distress that are rooted in experiences of loss, displacement, and structural problems in the host country, such as discrimination (Kirmayer et al., 2014), socioeconomic isolation, and dominant discourses of victimhood and Islamophobia. The understanding that these may be important causes of mental health problems among refugees calls for more research to add to the existing literature on these post-migration stressors and their effects.

Syrian refugees occupy an in-between space in which they build on their Syrian background and simultaneously reconstruct and adapt their identities to the Dutch context. Part of this context are the dominant societal discourses on Syrian refugees, which contribute to them being seen, and seeing themselves, as violent terrorists or weak victims (see also Van Es et al., 2021), and generally as people in need of (professional) help, images that clash with their perceived self from the past. Through our analysis of the hardships young Syrian refugees face and the ways in which they deal with them, it has become clear that the main problem for refugees is that they struggle with a fragmentation of their identity as a result of displacement and these dominant societal discourses.

In struggling to reconcile their fragmented identities, Syrian refugee youths and their parents engage with memories. Our findings demonstrate that our participants embrace two simultaneous strategies despite their apparent opposition: the remembering of some memories, and the active forgetting of, or not dwelling on, others. This finding is in accordance with the study of Gemignani (2011), who found that refugee participants from the former Yugoslavia adopted two main narrative strategies (“the past is past” and “the past is our strength”), and that these strategies help refugees attain individual and collective identity-making. Similarly, Barakat and Philippot (2018) and Puvimanasinghe et al. (2015) concluded that articulating empowering narratives of the past and silencing other narratives may generate the reconstruction of new and less shattered individual and collective identities for the refugees they studied.

Conversely, some of our respondents used adverse experiences of hardship and distress as sources of strength. Counter to what might be expected, they selectively remember memories of stressful and traumatic events because that helps them feel that they are a strong survivor. Similarly, Barakat and Philippot (2018), who analyzed the narratives of Syrian women in Lebanon, found that they embrace hardships as a foundation of resilience, redefining the traumatic event and ascribing value to loss as a way of giving meaning to their current realities. Similarly, Shakespeare-Finch et al. (2014) found that Burmese refugees narrated their traumatic event as a source of personal strength. Selectively forgetting is as important as selectively remembering, because silence may function as a way to generate self-worth (Ghorashi, 2008). Indeed, De Haene et al. (2018) urged therapists to “acknowledge the role of silence as an active position in coping with trauma, in which survivors reclaim power and mobilize an inversion of the helplessness invoked by traumatic life events.” Hjern and Jeppsson (2005) go even one step further, stating that the basic assumption underlying “most PTSD-centered mental health care” of “working through” distressful experiences is influenced by Christianity and does not fit with forgetting as a means of coping in various other cultures.

The design of this study means that we cannot conclude whether avoiding or silencing memories is the best way to prevent or deal with mental health problems for (these) Syrian refugees, now or in the future, nor whether standard methods for dealing with PTSD should be adjusted. We did, however, show how the refugees we spoke with struggle with the dominant discourse of themselves as weak victims, something that may be enforced by treatments that ask them to focus on memories that make them feel disempowered. Instead, the refugees we spoke with want to forget both some positive memories, which remind them of what they are missing in the present, as well as negative memories, because these hinder their focus on their future. We have also shown how this process of forgetting is complicated because of the social–economic and legal hardships and isolation they currently face, which makes it hard to distract themselves.

## Conclusion

In the Introduction, we described how identities partially result from belonging to a group and being ascribed group characteristics by the dominant narratives in society. Based on the narratives of the survivors we interviewed, we argue that their narratives of their past are truly counter-narratives, narratives that counter both individual stories of them as weak incompetent victims or violent terrorists and that counter the collective stories of the Syrian refugees’ as victims and/or perpetrators. Similarly, Barakat and Philippot (2018) pointed to the relevance of centering a collective identity for some Syrian refugees, which they called defining a social and political activist motivation to keep going. Our article therefore contributes to the literature exploring how refugees use stories to reconstruct the past and create their present individual identities, as well as their collective identity (Bartholomew et al., 2015; Puvimanasinghe et al., 2015; Ramsden & Ridge, 2013). The counter-narratives of Syrian refugees, in which they build up an alternative meaning of being a Syrian refugee (e.g., a strong survivor, rather than an aggressive victim) can be seen as more than a way of dealing with individual psychological problems. They may contribute to more positive perceptions of (Syrian) refugees in general and potentially a less-fragmented sense of self for the refugees themselves.

Our analysis represents a critical perspective that is relevant to scholars, practitioners, and policymakers concerned with refugees’ reception and inclusion. Whereas the conventional literature and popular media describe (Syrian) refugees as victims who need to deal with the traumatic events they experienced before and during their flight, the Syrians we spoke with expressed that their main challenges were having to deal with hardships in their present and in their host country. They particularly struggled with the dominant discourses of refugees “in need of help,” which makes them feel weak. The refugees countered these discourses by telling collective and individual stories of being strong and competent, including during traumatic events in their past. The youths and the parents we interviewed presented individual and collective counter-narratives of Syrians and refugees as being strong, resilient, and capable people who are oriented toward the future and who try to forget elements of their past.

Both mechanisms to deal with their hardships, telling the (individual and collective) counter-narrative of Syrians as strong and competent and the mechanism of forgetting what made them feel weak in their past, are alternative explanations for why these refugees do not seek professional psychosocial help. Seeking professional help would mean giving in to the dominant discourse of Syrians as victims, and professional (mental health) treatment would mean paying (more) attention to the exact memories they want to forget. The processes of creating meaning through narratives is a way to make sense of their life experiences, and this way may not fit with the idea of seeking professional help, at least not help that regards them as victims. Our finding that Syrian refugee youths use the counter-narratives of being strong and competent to deal with their experience of identity fragmentation in a context of displacement and dominant discourses of refugees as “in need of help” offers an alternative explanation for why these refugees do not seek professional help in dealing with their hardships.
